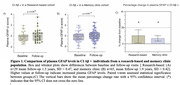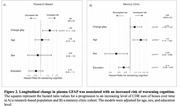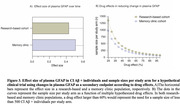# Exploring the utility of Plasma GFAP as a secondary endpoint in Alzheimer's disease Clinical trials to monitor disease progression

**DOI:** 10.1002/alz.093417

**Published:** 2025-01-09

**Authors:** Sarah Abbas, Pamela C.L. Ferreira, Bruna Bellaver, Guilherme Povala, Francieli Rohden, Cristiano Schaffer Aguzzoli, Hussein Zalzale, Guilherme Bauer‐Negrini, Carolina Soares, João Pedro Ferrari‐Souza, Douglas Teixeira Leffa, Firoza Z Lussier, Marina Scop Madeiros, Matheus Scarpatto Rodrigues, Markley Oliveira, Cynthia Felix, Cécile Tissot, Joseph Therriault, Andrea L. Benedet, Nicholas J. Ashton, Ann D Cohen, Oscar L. Lopez, Dana Tudorascu, Victor L Villemagne, Anum Saeed, Helmet T. Karim, Chang Hyung Hong, Hyun Woong Roh, Thomas K Karikari, Henrik Zetterberg, Kaj Blennow, Eduardo R. Zimmer, Pedro Rosa‐Neto, Sang Joon Son, Tharick Ali Pascoal

**Affiliations:** ^1^ University of Pittsburgh, Pittsburgh, PA USA; ^2^ Department of Psychiatry, University of Pittsburgh School of Medicine, Pittsburgh, PA USA; ^3^ Universidade Federal do Rio Grande do Sul, Porto Alegre, Rio Grande do Sul Brazil; ^4^ Brain Institute of Rio Grande do Sul, PUCRS, Porto Alegre, RS Brazil; ^5^ Federal University of Rio Grande do Sul, Porto Alegre, RS Brazil; ^6^ Translational Neuroimaging Laboratory, The McGill University Research Centre for Studies in Aging, Montréal, QC Canada; ^7^ Lawrence Berkeley National Laboratory, Berkeley, CA USA; ^8^ University of Gothenburg, Gothenburg Sweden; ^9^ University of Gothenburg, Mölndal, Gothenburg Sweden; ^10^ University of Pittsburgh Alzheimer's Disease Research Center (ADRC), Pittsburgh, PA USA; ^11^ Ajou University School of Medicine, Suwon Korea, Republic of (South); ^12^ Ajou University School of Medicine, Suwon, Gyeonggido Korea, Republic of (South); ^13^ University of Pittsburgh School of Medicine, Pittsburgh, PA USA; ^14^ Department of Psychiatry and Neurochemistry, Institute of Neuroscience and Physiology, The Sahlgrenska Academy, University of Gothenburg, Mölndal, Gothenburg Sweden; ^15^ Ajou University Hospital, Suwon Korea, Republic of (South)

## Abstract

**Background:**

Recent anti‐amyloid clinical trials have incorporated plasma glial fibrillary acidic protein (GFAP) as an exploratory endpoint, reporting a notable decrease in plasma GFAP levels over time. Additionally, plasma GFAP has been associated with Aβ pathology and cognitive decline in individuals with cognitive impairment, making it a robust biomarker of neuroinflammation for Alzheimer’s disease (AD). Here, we tested the utility of changes in plasma GFAP as a secondary endpoint in AD clinical trials focusing on cognitively impaired (CI) individuals.

**Method:**

We evaluated CI Aβ+ individuals from two distinct cohorts: TRIAD (n=29, mean age (SD) = 71.5 (6.48), %MCI=44.8 and BICWALZS (n=65, mean age (SD) = 71.5 (6.48), %MCI=66.1]. All individuals had Aβ PET measurements at baseline. Paired t‐tests compared plasma GFAP levels between baseline and follow‐up. Cox proportional‐hazards analysis was performed to assess whether changes in plasma GFAP levels were predictive of increased odds for increasing CDR sum of boxes score over time. Sample size estimation was based on a hypothesized 25% drug effect on plasma GFAP reduction.

**Result:**

We found a significant increase in plasma GFAP levels compared to baseline in both cohorts (Figures 1A and 1B). The percentage change in the research‐based cohort was slightly higher, likely attributed to a higher proportion of AD individuals compared to the memory clinic cohort (Figure 1C). Longitudinal plasma GFAP was associated with an increased risk of worsening cognition over time (Figures 2A and 2B). The effect size was slightly higher in the memory clinic cohort likely due to a relatively smaller standard deviation (Figure 3A). Inclusion of CI Aβ + individuals in a clinical trial testing a hypothesized 25% drug effect would necessitate 1354 participants in the research‐based cohort and 977 participants in the memory clinic cohort per study arm (Figure 3B).

**Conclusion:**

Changes in GFAP are associated with worsening cognition over time in CI Aβ + individuals. Our findings suggest Plasma GFAP as a robust biomarker to be used as a secondary endpoint to monitor disease progression in CI Aβ + in AD clinical trials. Our findings, replicated in both research and clinical settings, may have implications in AD clinical trials composed of a real‐world population.